# Preparing the Matrix

**Published:** 1906-03

**Authors:** Lee K. Stewart

**Affiliations:** Chicago.


### PREPARING THE MATRIX.

By Lee K. Stewart, D. D. S., Chicago.

In preparing the matrix for a porcelain inlay, the important consideration is accuracy. In all small cavities this is best obtained by burnishing the gold or platinum directly upon the tooth.

In many of the more difficult cases, the operator had best do this too, but if desirable, an impression of the cavity can be taken with a quick setting cement, gutta percha or modelling compound, this impression imbedded in plaster, and a model made with cement or amalgam. If cement is used for the impression, talcum powder or soap-stone must be rubbed over the cavity, so that the impression will come away easily; and they must be used too if a cement model is to be made from a cement impression. If amalgam is used in making a model from a cement impression, it is well to immerse the cement and amalgam mass in aqua-ammonia for several hours, to prevent fracture of the amalgam in separating.

Before the next sitting the platinum or gold is fitted to this model and this can best be done with the model of the cavity placed in the cup of the Brewster Matrix Press, and the metal gradually forced into position with burnishers and finished under pressure in the press. The fitting of the matrix is then easily completed by burnishing it directly into the tooth cavity. The drawing of a strip of rubber dam or tape over the matrix in position, will aid in holding the metal and the use of serrated amalgam pluggers as burnishers, with absorbent cotton, or spunk, will facilitate the operation.

The experienced inlay operator prepares almost all matrices directly upon the cavity, being careful to first carry the metal into the deepest part and afterward burnishing out any folds in the border. Nevertheless it is sometimes better both because of the nature of the cavity. and for want of time. to take an impression and proceed as suggested, and I prefer gutta percha for the impression and amalgam for the model.

After several years' experience I am convinced that for all but some very large restorations in the bicuspids and molars. the best results are obtained by finishing the fitting of the matrix directly upon the tooth itself. If the matrix is to be completed upon the model, cement should be used for the impression and cement or amalgam for the model, and the matrix made with a press as before described.

